# From islands to landscapes: Integrating genomic and advanced eco-geographic tools in area-wide tsetse control

**DOI:** 10.1371/journal.pntd.0014752

**Published:** 2026-10-09

**Authors:** Norah Saarman, Chantel Janet De Beer, Philippe Solano, Antoine Barreaux, Geoffrey Gimonneau, Sophie Ravel, Robert Opiro, Giuseppe Amatulli, Giuliano Cecchi, Serap Aksoy, Brian Weiss, Kostas Bourtzis, Adly M. M. Abd-Alla

**Affiliations:** 1 Department of Biology, Utah State University, Logan, Utah, United States of America; 2 Insect Pest Control Section, Joint FAO/IAEA Centre of Nuclear Techniques in Food and Agriculture, Department of Nuclear Sciences and Applications, International Atomic Energy Agency, Vienna, Austria; 3 INTERTRYP, CIRAD, IRD, and University of Montpellier, Montpellier, France; 4 INTERTRYP, CIRAD, Nairobi, Kenya; 5 International Centre of Insect Physiology and Ecology (ICIPE), Nairobi, Kenya; 6 Department of Biology, Gulu University, Gulu, Uganda; 7 Spatial Ecology, Penn, United Kingdom; 8 Animal Production and Health Division, Food and Agriculture Organization of the United Nations (FAO), Rome, Italy; 9 Yale School of Public Health, New Haven, Connecticut, United States of America; Hokkaido University International Institute for Zoonosis Control, JAPAN

## Abstract

Tsetse (Diptera: Glossinidae) are the exclusive cyclical vector of human and animal African trypanosomoses and continue to threaten health and agricultural productivity across sub-Saharan Africa. Area-wide integrated pest management (AW-IPM) strategies provide a coordinated framework for combining tools such as the sterile insect technique (SIT), insecticide-treated targets and cattle, and others. This review examines the success and challenges of AW-IPM in tsetse control and advances in population genetics, species distribution modeling, and landscape genetics that improve identification of candidate populations for optimized AW-IPM design. We introduce the Coordinated Research Project (CRP) “Tsetse Population Genetics,” supported by the Joint FAO/IAEA Centre, which aims to develop standardized tools and data to guide SIT within integrated control strategies. We identify key gaps in evidence for population isolation and reinvasion risk and propose an integrated, decision-ready framework. The framework combines updated species distribution models with scalable genomic tools to generate connectivity maps and, where feasible, source–sink dynamics, to prioritize intervention sites and distinguish post-control reinvasion from true elimination.

## Author summary

Tsetse flies transmit the parasites that cause sleeping sickness in people and African animal trypanosomosis in livestock. Although the number of human cases has declined substantially, these diseases still threaten health, food security, and rural livelihoods across much of sub-Saharan Africa. Control programs using area-wide integrated pest management (AW-IPM), including tools such as the sterile insect technique and insecticide-based methods, have achieved important successes. However, long-term success depends on whether local tsetse populations are truly isolated or can be reinvaded from surrounding areas.

New advances in genetic analysis and spatial mapping now allow researchers to better understand where tsetse populations occur, how they are connected, and how they move across landscapes. In this review, we show how combining these tools can improve planning, help identify areas where elimination is realistic, and strengthen monitoring after control efforts. We argue that the main challenge is not a lack of technology, but the need to apply these approaches consistently and link scientific evidence more directly to control decisions.

## 1. Introduction of the challenges posed by tsetse

African trypanosomoses are parasitic diseases caused by protozoa of the genus *Trypanosoma* that are mainly transmitted by blood-feeding tsetse (Diptera, Glossinidae), the sole cyclical vectors of these parasites [1,2]. The diseases affect both humans (human African trypanosomiasis, HAT) and animals (African animal trypanosomosis, AAT) [3,4]. HAT comprises two forms: Gambian sleeping sickness (g-HAT), caused by *Trypanosoma brucei gambiense* and endemic to West and Central Africa, Rhodesian sleeping sickness (r-HAT), caused by *T. brucei rhodesiense* endemic to eastern and southern Africa [5–8]. HAT reported incidence declined from almost 40,000 cases annually in the late 1990s to fewer than 1,000 cases since 2019 [9]. The World Health Organization (WHO) roadmap till 2030, targets the interruption of transmission for g-HAT and elimination as a public health problem for r-HAT (fewer than one case per 10,000 inhabitants per year per health district) [9].

AAT remains a major constraint on livestock production and agricultural development across large areas of sub-Saharan Africa [10]. An estimated 100 million cattle and even more small ruminants are at risk [10,11]. Direct annual livestock losses are estimated at US $1.2 billion, increasing to approximately US $4.75 billion when indirect costs are included [12,13]. At least 35 million doses of trypanocidal drugs are administered annually at a cost of approximately €30 million [14]. To address these challenges, a progressive control pathway (PCP) for AAT and a phased conditional approach (PCA) for tsetse management have been proposed [15–17], emphasizing context-specific strategies for suppression or eradication based on environmental, epidemiological, and socioeconomic conditions.

Within this framework, the Coordinated Research Project (CRP) on tsetse population genetics, sponsored by the Joint FAO/IAEA Centre of Nuclear Techniques in Food and Agriculture, aims to strengthen the scientific basis for area-wide integrated pest management. CRPs provide structured collaboration among Member State institutions to advance methods and translate research into operational tools. The present CRP engages 19 institutions contributing sampling, genotyping, modeling, and validation efforts, supported by research coordination meetings and capacity-building activities. Its core objectives and deliverables are summarized in Table 1 and reflect recurring evidence gaps in isolation, reinvasion, and decision thresholds across AW-IPM settings. We therefore conducted a targeted review spanning operational interventions and enabling genomic and spatial methods, as described below.

**Table 1:** Expected outputs and outcomes from the Coordinated Research project “Tsetse Population Genetics and the Sterile Insect Technique: Bridging the Gap for Effective Vector Control”.

Specific objective	Deliverables
1. Develop and validate species distribution models (SDM) for SIT-targeted species	SDM at 1 km resolution; validated models for key *Glossina* species
2. Design and validate identification tools for SIT applications	Molecular markers for species/subspecies identification; markers to distinguish released SIT males from wild males
3. Determine population structure and gene flow to assess isolation	Target sampling sites identified; field samples and metadata in open database; genotyping and bioinformatics outputs; genetic structure and connectivity models
4. Integrate SDM and population genetics outputs to identify SIT candidates	Combined distribution and connectivity maps; identification of candidate isolated ‘tsetse islands’; assessment of control program impacts on population structure

## 2. Methods

This review synthesizes current knowledge, identifies key methodological gaps, and outlines a coordinated genomic and ecological framework to support evidence-based vector control and sustained progress toward the control and elimination of human and animal African trypanosomosis. We conducted a targeted literature review focused on (i) area-wide integrated pest management (AW-IPM) approaches for tsetse control (including SIT, insecticide-treated targets/traps/livestock), and (ii) methods and applications of population genetics, species distribution modeling, and landscape genetics relevant to identifying candidate populations and reinvasion pathways. Literature was identified through iterative searches of Web of Science, Scopus, PubMed, and Google Scholar using combinations of keywords such as “tsetse,” “Glossina,” “African trypanosomiasis,” “area-wide integrated pest management,” “sterile insect technique,” “sequential aerosol technique,” “Tiny Targets,” “population genetics,” “landscape genetics,” “species distribution model,” “habitat suitability,” and “connectivity,” supplemented by backward and forward citation tracking of key reviews and influential primary studies. We primarily considered peer-reviewed articles, technical reports, and policy-relevant syntheses produced by major public health and agriculture organizations published between 1990 and 2025, but also included earlier landmark studies where appropriate. We prioritized studies that (a) included field-deployed interventions or operational planning components, (b) generated genetic evidence of population structure/connectivity or methods directly applicable to control design, or (c) developed or validated species distribution models (SDMs)/connectivity models using georeferenced occurrence and environmental data. For inclusion, studies needed to report sufficient methodological detail to evaluate relevance and transferability (e.g., sampling design, marker type, model validation, and geographic context). Evidence was synthesized qualitatively by ecological group (riverine, savannah, forest) and by methodological themes, highlighting areas of convergence, persistent limitations, and opportunities for standardization across sub-Saharan Africa.

## 3. Lessons from existing AW-IPM programs

AW-IPM integrates multiple control tactics to suppress or eliminate pest populations across defined geographic areas [18,19]. Tsetse control has evolved from last century early habitat management and insecticide campaigns [20–24] to more targeted approaches including insecticide-treated traps and targets [25,26], insecticide-treated cattle [27], odor-based control [24,28], sequential aerosol technique (SAT) [20,29] and the sterile insect technique (SIT) [30–32]. Each tool presents tradeoffs in cost, scalability, infrastructure, and permanence [18,33–38]. These tradeoffs are summarized in Table 2, while Box 1 highlights representative examples of how ecological context shaped outcomes.

**Table 2:** Strategic comparison of key AW-IPM tools for tsetse control.

Control Tool	Best Ecological Context	Primary Objective	Strengths or Advantages	Key Operational Constraints	References
Habitat Management	Agricultural margins; managed landscapes	Long-term suppression through habitat modification	Long-term effectiveness	Labor-intensive; potential land-use tradeoffs, non-target effects (pollinators and more)	[22,24]
Insecticide-Treated Livestock	Mixed livestock–vector systems; savannah and peri-domestic areas	Suppression with livestock protection co-benefits	Cost-effective, especially with farmer participation	Consistent treatment coverage; farmer participation; high livestock density; can increase insecticide resistance in ticks	[22,23]
Insecticide-Treated Targets (including tiny targets)	Riverine or woodland habitats; high vector–human or vector–livestock contact zones	Sustained suppression and prevention of humans from infective tsetse bites	Short-term effectiveness, high target specificity and minimal effects on non-target insects	Continuous deployment required; reduced effectiveness in open savannah systems	[24,26,28,39,40]
Sterile Insect Technique (SIT)	Geographically or genetically isolated/ semi-isolated populations	Potential local eradication	Species-specific control with no impact on non-target insects	High logistical and financial cost; requires mass rearing, irradiation, sterile-to-wild ratio requirements, reproductive compatibility, strong baseline ecological and genetic data, suppression activities	[18,33–38]
Sequential Aerosol Technique (SAT)	Large, contiguous landscapes; savannah or mixed habitats with moderate–high tsetse density	Potential local eradication	Short-term effectiveness, rapid results	High logistical and financial cost; requires coordinated aerial deployment; weather-dependent; impact on non-target insects; needs integration with follow-up control to prevent reinvasion	[20,29]

Insecticide-treated traps and targets, particularly tiny targets, which are small, insecticide-treated blue-and-black fabric panels designed to attract and kill riverine tsetse flies, are key vector control tools in riverine systems [24,26,39,41]. Tiny targets have been successfully used to interrupt transmission of g-HAT and even elimination of some tsetse populations [40]. Related approaches such as insecticide-treated livestock [27] and emerging odor-based control [24,28] do not have documented success stories of long-term elimination. These trap- and target-based techniques provide cost-effective and scalable suppression but require sustained deployment and are less effective in open savannah habitats.

In open savannah habitats, SAT and SIT have been found to be more effective. SAT has been effective when GPS-guided and combined with treated barrier zones to prevent re-invasion [20]. SIT is similar to SAT in its ability to achieve local eradication in isolated or semi-isolated populations [25,31,38,42]; in more connected systems, it must be integrated with other suppression tools and barrier strategies such as sequential “rolling carpet” approaches [18,43]. However, SIT has an additional limitation in that it involves mass-rearing facilities, sterilizing males using ionizing radiation, and systematic release in large numbers to outcompete wild males and reduce fecundity in wild populations [18,31,32,44,45]. Therefore, SIT’s success also depends on high sterile-to-wild male ratios, reproductive compatibility, and strong baseline ecological and connectivity data [33–37].

Successful deployment depends on accurate knowledge of habitat suitability, spatial connectivity, and population structure [33,46–52]. For SIT, reproductive compatibility between released and wild populations is critical [19]. Although molecular tools based on microsatellites, mitochondrial DNA, and *Wolbachia* infection exist [53,54], additional tools are required for reliable subspecies differentiation [32,53,55]. Ecological and genetic data are therefore foundational to AW-IPM design and sustainability [36].

**Box 1. Area-wide integrated pest management (AW-IPM) success stories**

Isolation is one of the strongest predictors of AW-IPM success. When populations are geographically and genetically isolated, the risk of reinvasion is low, and suppression or eradication becomes more feasible [32,43–45]. This has been demonstrated in the most successful SIT-based, SAT-based and tiny target-based tsetse elimination campaigns to date:

**Table:** 

Location/ Context	Strategy	Key Outcome	References
**Unguja Island** **(Zanzibar)**	Sequential suppression (trapping + insecticide) followed by the Sterile Insect Technique (SIT)	*Glossina austeni* eradicated 1994–1997; no reinvasion detected	[42]
**Niayes Region,** **Senegal**	Stratified sampling, SDM-guided planning, genetic verification of isolation, and integrated SIT	*G. p. gambiensis* eradicated (2005-2022); African animal trypanosomosis eliminated in a major livestock zone	[31,32,56]
**Chad,**	Tiny targets in riverine habitats integrated with active screening and treatment	Substantial reductions in *Glossina fuscipes* populations (2014-2025) and marked decline in HAT incidence; cost-effective maintenance feasible	[40]
**Botswana**	Sequential aerosol technique (SAT) implemented using a rolling-carpet strategy with treated barrier zones in an isolated population	Local eradication of tsetse achieved (2001-2002) without reinvasion, demonstrating the importance of geographic isolation and coordinated area-wide implementation	[20,29]

## 4. Scientific foundations for integrating genomic and spatial methods

The methodological advances summarized in Box 2 have transformed our ability to characterize tsetse population structure, dispersal barriers, and reinvasion risk across heterogeneous landscapes [26,44,57–59]. Collectively, these developments provide the scientific basis for surveillance, intervention prioritization, and refinement of AW-IPM strategies [32,55]. More importantly, they now enable the integration of genomic and spatial inference into unified, decision-relevant frameworks capable of distinguishing isolated populations from connected systems and predicting reinvasion pathways.

**Box 2. Methodological advances enabling integrated tsetse genomics and spatial modeling**

**Genomic and molecular advances**

- **Microsatellites and sequence-based microsatellite genotyping (SSRseq; simple sequence repeat sequencing).** Microsatellites remain widely used for historical comparison and operational monitoring of tsetse populations. SSRseq replaces fragment-length scoring of microsatellites with direct sequencing of repeat regions, improving allele resolution, reproducibility across laboratories, and compatibility with high-throughput workflows.
- **Reduced-representation genome-wide sequencing (ddRAD, bestRAD, RAD-cap).** Reduced-representation approaches provide scalable, cost-effective genome-wide genotyping that enables high-resolution inference of population structure and connectivity, serving as a bridge toward whole-genome analyses. Their cost-effectiveness arises from targeting a consistent subset of the genome across many individuals, which reduces sequencing and data-processing costs per sample. This makes them well suited for both research applications such as landscape genetics requiring large sample sizes, as well as operational contexts such as surveillance or routine monitoring where budgets and turnaround time are limiting factors
- **Whole-genome sequencing and long-read platforms.** High-quality reference assemblies and structural variant resolution now permit fine-scale inference of demographic history, inbreeding, and genomic differentiation across ecological groups.
- **Target enrichment and low-input amplification workflows.** Capture-based enrichment strategies combined with low-input library preparation enable recovery of high-quality genomic data directly from field-preserved specimens, reducing reliance on laboratory colonies and minimizing dissection-intensive workflows.
- **Integrated vector–parasite–symbiont genomic recovery.** Whole-body sampling preserved in ethanol or RNA-stabilizing buffers allows simultaneous recovery of vector, parasite, and microbial DNA from single extractions, enabling multi-level eco-genomic analyses of transmission systems. This approach reduces reliance on specialized collection protocols and field dissections, minimizes contamination introduced during tissue handling, and improves reproducibility across sampling sites.

**Spatial and connectivity modeling advances**

- **High-resolution environmental data streams.** Remotely sensed climatic, land cover, and hydrological datasets now provide finer spatial and temporal resolution for habitat suitability modeling.
- **Continental occurrence synthesis (FAO tsetse atlas).** The FAO continental atlas provides an essential, standardized foundation of georeferenced presence records across sub-Saharan Africa [60] which is enhanced by a steadily increasing number of national atlases [61–63]. These occurrence data enable integration with climatic, land-use, and topographic layers to generate predictive habitat suitability models and guide targeted sampling.
- **Machine-learning–enhanced species distribution modeling.** Building upon atlas-based occurrence data, machine-learning frameworks (e.g., ensemble tree methods and neural networks) improve flexibility in modeling nonlinear ecological relationships while allowing explicit correction for sampling bias and spatial autocorrelation [14,64]. Careful attention to data quality, background selection, and spatial resolution remains essential for robust inference.
- **Resistance-surface and connectivity modeling with Machine-learning integration.** Machine-learning frameworks allow nonlinear modeling of ecological–genetic relationships, improving inference of dispersal barriers, movement corridors, and potential source–sink dynamics.
- **Integrated SDM–genomic frameworks**. Joint analysis of habitat suitability and genetic connectivity enables identification of isolated “tsetse islands,” reinvasion corridors, and spatially explicit intervention targets.

Genetic analyses have long informed population structure, gene flow, and vector monitoring [35,65,66]. Traditional morphology-based identification remains problematic for closely related taxa [53,67], and although molecular tools have been developed [53,68], scalable and standardized solutions are still needed. Microsatellites, geometric morphometrics, and reduced-representation sequencing approaches such as ddRADseq and SSRseq have improved resolution of genetic structure and connectivity [67–79]. These studies reveal contrasting spatial dynamics across ecological groups: riverine species often show structure aligned with river corridors [33,46,80,81], savannah species tend toward broader connectivity with fragmentation driven by land-use change [71,74,82,83], and forest species remain understudied but appear more isolated [84]. These patterns directly inform identification of candidate populations for AW-IPM.

SDMs provide complementary spatial insight by predicting habitat suitability and guiding targeted sampling [34,44,78,85–88]. Advances in geographic information system (GIS) [89], machine-learning approaches [86], and expanded occurrence datasets such as the FAO continental atlas [60] enable higher-resolution and bias-aware predictions. Tsetse distributions are shaped by climate, land cover, ecological interactions including host availability, pathogens and symbiotic bacteria and dispersal corridors [33,46,90,91], all of which are shifting due to land use and climate change [33,46,92]. Validated SDMs therefore play a critical role in identifying persistent habitats, reinvasion risks, and priority intervention zones.

Landscape genetics integrates genetic and environmental data to model resistance surfaces and quantify how landscape features influence gene flow [44,46,93,94]. Machine-learning frameworks improve modeling of nonlinear ecological–genetic relationships and relax assumptions inherent in traditional approaches [83]. When combined with SDMs, connectivity models can identify isolated “tsetse islands,” dispersal corridors, and potential source–sink dynamics relevant to intervention planning [44,46,95]. Here, “tsetse islands” refer to geographically or ecologically isolated populations with limited gene flow, dispersal corridors are landscape features that facilitate movement between populations, and source–sink dynamics describe situations where high-quality habitats (sources) sustain net population growth and export individuals to lower-quality habitats (sinks) that would not persist without immigration.

The conceptual and operational progression of these approaches is illustrated in the foundational studies highlighted in Box 3, which span classic SIT eradication in isolated systems, genetics-informed strategy selection, spatial connectivity modeling, tiny target implementation, and machine-learning integration. Despite these advances, implementation remains fragmented. Most studies remain localized or species-specific [37,48,50,96,97], and continental-scale integration of SDMs and genetic connectivity is limited outside a few well-characterized systems [33,98]. A unified framework that standardizes sampling, genotyping, connectivity modeling, and post-control monitoring is therefore needed to translate these advances into operational AW-IPM decision-making [36]. The following section outlines key research priorities to achieve this integration.

**Box 3. Five foundational papers shaping modern AW-IPM in tsetse control**

1. **Vreysen et al. 1999 (Backhuys Publishers)**—Eradication of *Glossina austeni* on Unguja Island using the Sterile Insect Technique (SIT). Landmark demonstration of successful area-wide eradication in an isolated system [42].
2. **Solano et al. 2010 (PLoS NTD)**—Population genetics to select suppression versus eradication strategies in Senegal (Niayes). Established genetics as an operational decision tool in AW-IPM [32].
3. **Bouyer et al. 2015 (PNAS)**—Mapping landscape friction to identify isolated populations suitable for elimination. Introduced spatially explicit connectivity modeling into tsetse control planning [33].
4. **Kgori, et al. 2006 (Acta Tropica)**—The use of aerial spraying to eliminate tsetse from the Okavango Delta of Botswana. Successful elimination was attributed to adequate insecticide dose, GPS-guided precision flying, large-scale spray blocks, and treated barrier zones that prevented re-invasion [20].
5. **Bishop et al. 2021 (Evol Appl)**—Machine-learning integration of ecological and genetic data for spatially explicit vector control planning. Represents the transition toward AI-enabled eco-genomic integration [86].

## 5. Roadmap and research priorities for genomic and spatial integration

Despite substantial advances in population genetics, SDMs, and landscape genetics [26,44,57,59,86], their simultaneous application to tsetse control remains fragmented and scarce. Most studies have been conducted at local or national scales, often focusing on single species or discrete ecological settings [37,48,50,96,97], with only a few systems characterized in sufficient detail to link genetic structure, habitat suitability, and intervention outcomes [33,98]. As a result, datasets remain geographically patchy, methodological approaches vary across studies, and continental-scale integration across sub-Saharan Africa is limited. Post-control genetic monitoring is inconsistently implemented, reducing the ability to distinguish true eradication from temporary suppression or reinvasion [36]. A unified framework that standardizes sampling, genotyping, connectivity modeling, and spatial analysis across ecological groups is therefore needed to translate these scientific advances into durable, evidence-based AW-IPM strategies.

A first priority is the development of standardized, bias-aware species distribution models capable of supporting continental-scale integration across sub-Saharan Africa. The FAO continental tsetse atlas and the related national atlases provide a critical foundation of occurrence data [60,61], and advances in GIS and machine-learning approaches offer improved predictive capacity [86,89]. However, updated SDMs are needed that incorporate high-resolution environmental layers, explicitly address sampling bias, and are validated across representative species from riverine, savannah, and forest ecological groups [34,44,78,85–88]. Beyond predicting habitat suitability, such models should be explicitly linked to sampling design, reinvasion risk assessment, and long-term surveillance planning. Standardized SDM frameworks will improve comparability across regions, optimize allocation of limited field resources, and provide the spatial backbone necessary for integrating genomic and connectivity analyses. Such efforts are currently ongoing for continental modeling of tsetse species distribution aligned with AAT geospatial modeling [63] which is a key step in advancing our understanding of tsetse habitat suitability and will warrant continuous updating and tailoring to specific AW-IPM programs to provide key information at scale.

A second priority is the establishment of harmonized and scalable genomic infrastructure to resolve species identification, quantify population structure, and enable cross-regional connectivity analyses, while also enabling integrated vector–parasite–symbiont and functional genomic inference from field-collected specimens. Although microsatellites, morphometrics, and reduced-representation sequencing approaches such as ddRADseq and SSRseq have improved resolution of genetic structure in several systems [67–79], datasets remain geographically uneven and taxonomically incomplete [37,48,50,96,97]. Standardized genotyping frameworks applied across ecological groups and countries are required to enable direct comparison of population structure and gene flow across the tsetse belt. Emerging developments in whole-genome sequencing, target enrichment, and low-input library preparation now permit recovery of high-quality genomic data directly from field-preserved specimens, reducing dependence on laboratory colonies and minimizing the need for dissection-intensive workflows. Such approaches also create opportunities to recover vector, parasite, and symbiont DNA from single extractions, enabling integrated eco-genomic analyses of transmission systems. Coordinated sampling strategies informed by updated SDMs will be essential for filling geographic gaps and ensuring that genomic data contribute meaningfully to continental-scale inference.

A third priority is the integration of habitat suitability and genetic connectivity into unified, spatially explicit models capable of identifying isolated populations, dispersal corridors, and source–sink dynamics across sub-Saharan Africa (Fig 1). While SDMs predict potential occurrence and habitat persistence [34,44,85–88], genetic data reveal realized movement and gene flow [33,46,80,81,94,99,105–109]. Resistance-surface modeling and machine-learning frameworks now provide greater flexibility for capturing nonlinear ecological–genetic relationships and reducing restrictive assumptions inherent in earlier approaches [44,46,86,94,95]. However, continental-scale integration of these methods remains limited outside a few well-characterized systems [32,44], and robust inference of directional movement and source–sink structure remains challenging. Strengthening joint spatial–genomic modeling and implementing systematic post-control genetic monitoring will be essential for distinguishing true isolation from transient fragmentation and for anticipating reinvasion risk in dynamic landscapes [36].

**Fig 1:**
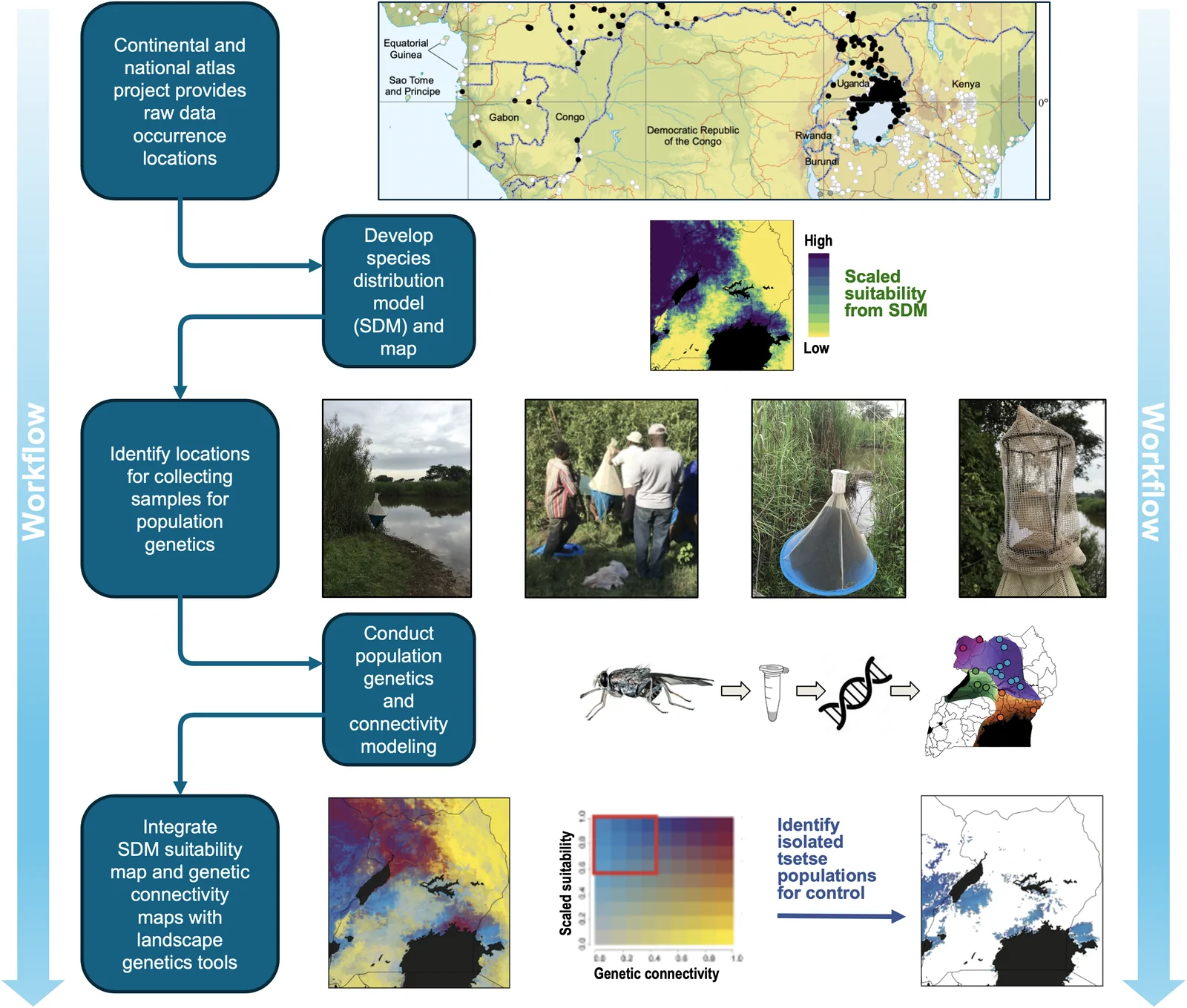
Integrated genomic and spatial workflow for identifying isolated tsetse populations for area-wide integrated pest management (AW-IPM). Continental and national occurrence records are first used to develop SDMs and identify environmentally suitable habitats for targeted field sampling. Genetic data collected from these populations are then analyzed to estimate population structure and connectivity. Finally, habitat suitability, genetic connectivity, and landscape resistance are integrated to distinguish isolated populations with high elimination potential from connected populations where reinvasion risk may require suppression or barrier strategies. This framework supports evidence-based planning and evaluation of AW-IPM interventions across sub-Saharan Africa. The continental occurrence map is adapted from the FAO Continental Atlas of the Distribution of Tsetse Flies in Africa [60], with permission. Field photographs are courtesy of Brian Weiss. The population genetics and landscape genetics maps were generated by the authors from publicly available data reported in [80,98–104].

Collectively, these priorities provide a roadmap for advancing standardized genomic and spatial approaches that can support evidence-based AW-IPM across sub-Saharan Africa. The broader policy implications and possible applications to operational decision-making are discussed below.

**Box 4. Key learning points**

1. **AW-IPM outcomes are context-dependent.** Isolation and reinvasion risk largely determine whether interventions can achieve elimination versus requiring sustained suppression and barrier strategies.
2. **Genomic data are operational.** Species/subspecies identification, population structure, and connectivity estimates directly inform intervention boundaries, compatibility considerations for SIT, and post-control monitoring.
3. **SDMs provide the spatial backbone.** Standardized occurrence resources (including the FAO continental tsetse atlas) coupled with bias-aware SDMs improve sampling design, surveillance targeting, and assessment of persistence and reinvasion risk.
4. **Landscape genetics links habitat to realized movement.** Resistance-surface and connectivity modeling identify dispersal corridors and candidate isolated populations, supporting prioritization and spatially explicit AW-IPM design.
5. **The main bottleneck is integration and standardization.** Harmonized sampling, genotyping, modeling workflows, and routine post-control genetic monitoring are required to move from case-by-case studies to durable, evidence-based AW-IPM at continental scale.

## 6. Conclusions and policy implications

Tsetse and the human and animal African trypanosomoses they transmit remain major constraints on health and development across sub-Saharan Africa. While tools such as the sterile insect technique (SIT) and insecticide-based methods when implemented in the frame of AW-IPM have delivered strong outcomes in specific ecological and operational contexts, durable success at scale depends on correctly defining intervention boundaries and reinvasion risk. In practice, this requires moving from assumptions about isolation based on geography or administrative borders toward evidence-based inference of habitat suitability, realized connectivity, and post-control movement and the probability of successful elimination.

Recent advances in population genomics, SDMs, landscape genetics and mathematical modeling now make this shift feasible. When implemented within harmonized sampling and analysis frameworks, these approaches can (i) identify candidate isolated “tsetse islands” where elimination is plausible, (ii) map dispersal corridors and likely reinvasion pathways where barrier strategies and sustained suppression are required, and (iii) provide standardized baselines for genetic monitoring to distinguish true elimination from temporary suppression or recolonization. Together, standardized SDMs, harmonized genomic datasets, integrated connectivity modeling, and tsetse elimination mathematical frameworks provide the foundation for moving beyond descriptive mapping toward predictive, spatially explicit inference of isolation, reinvasion pathways, and long-term population stability. These integrated approaches will improve the ability to distinguish true eradication from temporary suppression, anticipate ecological shifts driven by land-use and climate change, and design AW-IPM strategies that are resilient to dynamic landscapes while providing quantitative support for declaring elimination [40]. Key learning points are summarized in Box 4.

The Coordinated Research Project (CRP) provides an operational mechanism to implement this transition by standardizing field sampling, scalable genotyping, and integrated spatial–genomic modeling across ecological groups and Member State programs. By linking atlas-informed SDMs with harmonized genomic infrastructure and connectivity inference, AW-IPM planning can become explicitly spatial, predictive, and comparable across regions. Achieving this vision will require coordinated international collaboration, methodological standardization, open data sharing, and systematic post-intervention monitoring. The primary bottleneck is no longer a lack of tools, but the ability to deploy them consistently and interpret them within decision thresholds that support planning, implementation, and post-intervention verification.
